# Effect of workplace- versus home-based physical exercise on pain in healthcare workers: study protocol for a single blinded cluster randomized controlled trial

**DOI:** 10.1186/1471-2474-15-119

**Published:** 2014-04-07

**Authors:** Markus D Jakobsen, Emil Sundstrup, Mikkel Brandt, Anne Zoëga Kristensen, Kenneth Jay, Reinhard Stelter, Ebbe Lavendt, Per Aagaard, Lars L Andersen

**Affiliations:** 1 National Research Centre for the Working Environment, Lersø Parkalle 105, Copenhagen, Denmark; 2 Institute for Sports Science and Clinical Biomechanics, University of Southern Denmark, Odense, Denmark; 3 Coaching Psychology Unit, Department of Nutrition, Exercise and Sports, University of Copenhagen, Copenhagen, Denmark; 4 Electronics and Computer Science, Faculty of Physical and Applied Sciences, University of Southampton, Southampton, UK

**Keywords:** Musculoskeletal disorders, Occupational health, Health care, Strength training, Back pain, Neck pain, Shoulder pain

## Abstract

**Background:**

The prevalence and consequences of musculoskeletal pain is considerable among healthcare workers, allegedly due to high physical work demands of healthcare work. Previous investigations have shown promising results of physical exercise for relieving pain among different occupational groups, but the question remains whether such physical exercise should be performed at the workplace or conducted as home-based exercise. Performing physical exercise at the workplace together with colleagues may be more motivating for some employees and thus increase adherence. On the other hand, physical exercise performed during working hours at the workplace may be costly for the employers in terms of time spend. Thus, it seems relevant to compare the efficacy of workplace- versus home-based training on musculoskeletal pain. This study is intended to investigate the effect of workplace-based versus home-based physical exercise on musculoskeletal pain among healthcare workers.

**Methods/Design:**

This study was designed as a cluster randomized controlled trial performed at 3 hospitals in Copenhagen, Denmark. Clusters are hospital departments and hospital units. Cluster randomization was chosen to increase adherence and avoid contamination between interventions. Two hundred healthcare workers from 18 departments located at three different hospitals is allocated to 10 weeks of 1) workplace based physical exercise performed during working hours (using kettlebells, elastic bands and exercise balls) for 5 × 10 minutes per week and up to 5 group-based coaching sessions, or 2) home based physical exercise performed during leisure time (using elastic bands and body weight exercises) for 5 × 10 minutes per week. Both intervention groups will also receive ergonomic instructions on patient handling and use of lifting aides etc. Inclusion criteria are female healthcare workers working at a hospital. Average pain intensity (VAS scale 0-10) of the back, neck and shoulder (primary outcome) and physical exertion during work, social capital and work ability (secondary outcomes) is assessed at baseline and 10-week follow-up. Further, postural balance and mechanical muscle function is assessed during clinical examination at baseline and follow-up.

**Discussion:**

This cluster randomized trial will investigate the change in self-rated average pain intensity in the back, neck and shoulder after either 10 weeks of physical exercise at the workplace or at home.

**Trial registration:**

ClinicalTrials.gov (NCT01921764).

## Background

The socioeconomic cost in terms of sickness absence and poor work ability caused by musculoskeletal pain is substantial [1-9]. In general, occupations with high physical work demands show elevated prevalence of musculoskeletal pain [10] and long term sickness absenteeism [11].

Because the tasks of a healthcare worker are particularly physical demanding with awkward postures and high loadings on the back [12], the incidence of musculoskeletal pain is high. For example, among more than 8000 healthcare workers, 28%, 23% and 12% experienced severe pain in the neck/shoulders, low back and knees, respectively [9]. Further, the risk for long-term sickness absenteeism was increased by 47-92% when experiencing severe pain in these body regions [9]. Accordingly, strenuous perceived physical exertion during healthcare work is a risk factor for developing severe or chronic pain in the low back [13-15] and in the neck and shoulder [16-18]. This is not surprising considering biomechanical loadings during patient handling tasks frequently exceed the recommended safe limits for maximal acceptable compression forces on the back [19,20]. As healthcare work often is performed by women with low physical capacity, as manifested by low aerobic fitness and low muscle strength [21], the imbalance between physical work demands and physical capacity may lead to excessive loading of the musculoskeletal system [10], hence increasing the risk of overuse injury.

Although provision of manual handling equipment has increased the preventive efforts in the healthcare sector the incidences of musculoskeletal pain remains high [22]. This implies that ergonomic interventions aiming to reduce the physical demands and hence reduce or prevent the work-related musculoskeletal disorders might be insufficient when implemented as a single strategy [23]. An alternative strategy to prevent or reduce the work-related musculoskeletal disorders may be achieved by increasing the workers physical capacity through physical training interventions. Previous studies from our research group have shown promising and effective reductions in neck/shoulder/back pain in response to 10-20 weeks of strength training using kettlebells [24,25], elastic rubber bands [26,27] or free weight exercises [28-30] in laboratory technicians and office workers. However, as the working conditions of laboratory technicians and office workers chiefly comprises sedentary work and static activity of the neck/shoulder muscles, our previous positive findings may not be directly transferable to the working conditions of a healthcare worker often bending, turning and twisting during patient handling. Nevertheless, from a theoretical point of view, increasing physical capacity by means of on-site progressive physical training may provide an alternative way of reducing musculoskeletal pain in healthcare workers. On the other hand, healthcare workers are exposed to awkward postures, sudden loads and high lower back and neck and shoulder forces during patient handling that may hinder adequate recovery between the forceful tasks and the subsequent physical training sessions. The limited time per work task and stressful conditions experienced by modern-day healthcare workers, including shortage and handling of multiple tasks at the same time and frequent ad hoc tasks [31], of a healthcare worker may furthermore complicate the implementation of daily onsite physical training. The question remains whether physical training is a relevant and feasible intervention modality that, in addition to the on-going ergonomic interventions, efficiently can prevent and reduce musculoskeletal pain in healthcare workers.

Although the positive health promoting effects achieved through physical exercise is generally accepted, the implementation of physical workplace interventions is often met with low adherence [32]. Previous research from our research group indicate that external factors such as time, the exercise equipment accessibility and support from management and colleagues all play a significant role in the implementation and maintenance of physical conditioning at the workplace [33]. Performing physical exercise at the workplace together with colleagues may be more motivating for some employees thus increasing adherence. On the other hand, physical exercise at the workplace may be costly for workers and employers in terms of time spend and therefore result in decreased backup from unions and management, respectively. A key issue to increase adherence may be to focus on, not only, the physical exercises, but also the psychosocial work environment when implementing regular physical exercise at the workplace [34]. Thus, motivation of the participants and management through enhanced understanding of the physiological and social benefits as well as cost efficiency, in terms of i.e. reduced long term sickness, may be primary motivational factors. On the other hand, some people might feel uncomfortable when 1) leaving their colleagues behind to perform the exercises and/or 2) when exercising with colleagues and management. This may decrease adherence and therefore compromise the effects of the physical training compared to performing the exercises at home. It is therefore relevant to compare the efficacy of workplace- versus home-based training on musculoskeletal pain.

The aim of this study is to investigate the effect of workplace-based versus home-based physical exercise on musculoskeletal pain among healthcare workers. We hypothesize that supervised physical training at the workplace is superior to home-based exercise in reducing pain symptoms and increasing adherence.

## Methods/Design

### Trial design

A two-armed parallel-group, single-blind, cluster randomized controlled trial with allocation concealment is currently conducted among healthcare workers from 3 Danish hospitals. Clusters are hospital departments and hospital units. As each hospital department work together as a separate entity, cluster randomization is chosen to increase adherence and avoid contamination between interventions. The participants is allocated to a 10 week intervention period and paralleled assigned to receive either workplace-based or home-based physical exercise. The study duration is August 2013 to January 2014.

### Participants

Two hundred healthcare workers are recruited from 3 hospitals in Denmark. All participants are informed about the purpose and content of the project and have given their written informed consent to participate in the study, which was approved by the ethical committee (H-3-2010-062). All experimental conditions conformed to The Declaration of Helsinki.

### Recruitment

The recruitment was two-phased and consisted of a short screening questionnaire in June 2013 followed by a clinical examination and questionnaire in Aug-Sept 2013.

Firstly, in June 2013 a screening questionnaire was administered to 490 healthcare workers (aged 18-67 years) from three Danish hospitals. In total 314 replied to the questionnaire of which 275 were interested to participate in the research project. The initial inclusion criteria based on the screening questionnaire were female healthcare workers. Of the 275 interested respondents, 253 met the above criteria and were invited for a clinical examination in Aug-Sept 2013. Participants from the same hospital department and/or larger hospital units will be eligible for building a cluster.

A total of 207 employees were presented for the baseline clinical examination. Exclusion criteria were [1] hypertension (Systolic BP > 160, diastolic BP > 100), [2] a medical history of cardiovascular diseases (e.g. chest pain during physical exercise, heart failure, myocardial infarction and stroke) [3], traumatic or severe injury to the neck, shoulder, arm or hand regions [4], a medical history of life threatening disease, or [5] pregnancy.

**Figure 1:**
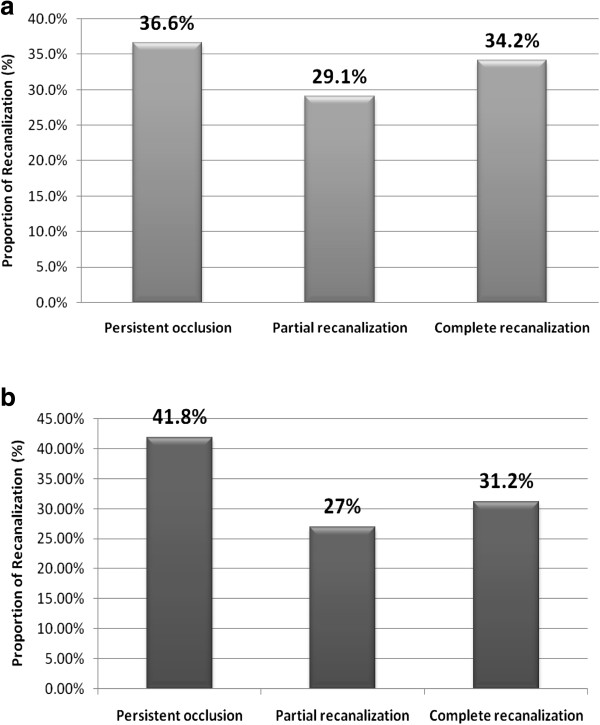
Flow-chart.

During the baseline clinical examination and questionnaire, 7 workers were excluded due to contraindications: 5 due to high blood pressure and 2 due to blood clot incidence within the last 2 years. The flow of participants is illustrated in Figure 1.

### Randomization

On the basis of the questionnaire we randomly allocated the 18 departments of 200 participants, using a computer-generated random numbers table (SAS), to either physical exercise at the worksite or at home. Subsequently, participants are informed by e-mail and by their management about group allocation. At the follow-up physical examination and questionnaire in Dec 2013-Jan 2014, all examiners will be blinded, and participants instructed not to reveal their particular intervention. Baseline characteristics and pain score of employees randomized into the two intervention groups are illustrated in Table 1. The participant’s average pain intensity in the lower back, neck and shoulder ranged from 0-9 (0-10 scale) at baseline.

**Table 1:** Characteristics of the two intervention groups

	**Physical exercise at home**	**Physical exercise at work**
N	89	111
Age (years)	44 (10)	40 *(12)
Height (cm)	168.0 (7.2)	168.4 (6.2)
Weight (kg)	68.9 (12.2)	67.5 (12.1)
BMI (kg∙m^-2^)	24.4 (4.0)	23.8 (3.8)
Average pain intensity in the back, neck and shoulders during the last week (scale 0-10)	3.10	3.02
Percentage of subjects with back pain intensity of at least 3 during the last week (scale 0-10)	57.3	59.5
Percentage of subjects with neck pain intensity of at least 3 during the last week (scale 0-10)	52.8	50.5
Percentage of subjects with shoulder pain intensity of at least 3 during the last week (scale 0-10)	58.4	51.4
Percentage of subjects with back pain for more than 30 days within the last year	20.2	36.0
Percentage of subjects with neck pain for more than 30 days within the last year	21.4	19.8
Percentage of subjects with shoulder pain for more than 30 days within the last year	28.1	25.2

Values are reported as Mean (SD).

*Difference between groups at baseline, P < 0.05.

### Interventions

The study aims to implement two comparable interventions for increasing the individual's physical capacity by means of physical exercise.

Participants in each cluster is allocated to a 10-week intervention period and paralleled assigned to receive either physical exercise at the hospital or physical exercise at home. Both training groups will perform physical exercises for 5 × 10 minutes a week. These two interventions are described below in detail.

### Physical exercise intervention at the worksite

Subjects randomized to this group (n = 111 subjects, n = 9 clusters) will perform supervised high-intensity strength training with elastic bands (Thera-Band®) and kettlebells during working hours at the hospital (their worksite). We have prioritized a training program design that is cost-efficient and involved easy-to-use exercises and training equipment, based on the assumption that subsequent post-intervention implementation at the worksite will only occur if the program is easily adopted, transparent and inexpensive to perform.

**Figure 2:**
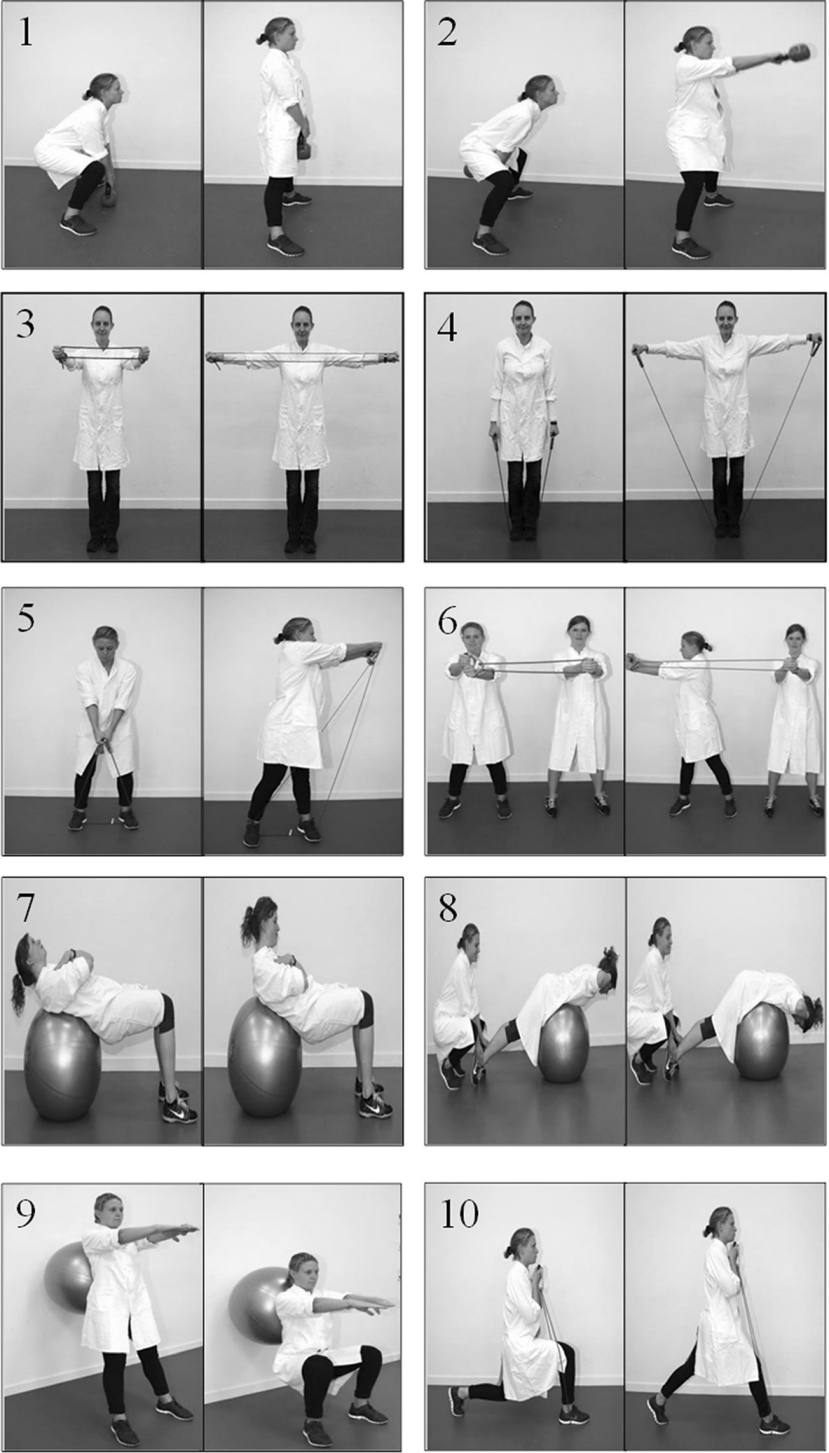
Exercises used in the physical exercise program at work: (1) deadlifts using kettlebell, (2) kettlebell swings, (3-6) squeeze, lateral raises, golf swings and woodchoppers using elastic tubing, (7-9) abdominal crunches, back extensions and squats using swiss ball, (10) lunges using elastic tubing.

The training program consists of 10 resistance exercises: [1] deadlifts using kettlebell [2], kettlebell swings [3-6], squeeze, lateral raises, golf swings and woodchoppers using elastic tubing [7-9], abdominal crunches, back extensions and squats using swiss ball [10], lunges using elastic tubing. All exercises are illustrated in Figure 2. We obtained consent from the people in Figure 2. For each training session the instructor will chose 4-6 exercises that are performed as circuit training i.e. quickly changing from one exercise to the next without pauses. The training sessions will comprise of 2 full circuits, and had a total duration of ~10 minutes. The instructors are exercise physiology students from University of Copenhagen, Denmark. The participants are encouraged to do the exercises together with their colleagues.

#### Training progression

Training intensity (loads) is progressively increased throughout the 10-week intervention period according to the principle of periodization and progressive overload [23]. The exercises woodchoppers, golf swings, lateral raises and lunges, are performed in a conventional manner using consecutive concentric and eccentric muscle contractions in a controlled manner. For these exercises relative loadings are progressively increased from 20 repetitions maximum (RM) at the beginning of the training period to 8 RM during the later phase.

All training sessions takes place in designated rooms located close to the worksite departments. All sessions will be supervised by a training instructor, who will instruct the participants how to perform the exercises, and help with exercise adjustment when needed. The instructors will focus on positive feedback and social engagement to maintain motivation throughout the intervention period.

#### Exercise adjustment

In case of acute worsening of pain or other contraindications during the time of training, the instructor will use the following 4-stage model to subsequently adjust the specific exercise.

Stage 1: Reduced loading intensity. A reduction in load (kg lifted or resistance of elastic tubing) is implemented in the specific exercise that may cause an increase in acute pain. A load reduction of up to 100% can be necessary, i.e. performing the movement without external resistance.

Stage 2: Reduced movement velocity. If a reduction in load fails to address the problem the movement velocity should be reduced.

Stage 3: Reduced range of motion (ROM). As a final action to solve the problem, the ROM should be reduced to cover a range where pain is not worsened. However, it is important not to decrease ROM too much since a reasonable part of dynamic strength training is desired interventional wise.

Stage 4: Interruption of the exercise. If none of the above stages is solving the problem, the specific exercise should be abandoned.

#### Coaching sessions

The main objective of including coaching was to reinforce the effects of physical training. The coaching intervention will involve the participants in a group dialogue, where the individuals are in focus and where all participants in a process of collaboration and co-creation will shape meaning and consider the value and meaning of their actions.

Meaning-making is considered to be one of the main purposes of facilitating the coaching dialogue [35,36] especially when it can happen collectively. Meaning is fundamental, because the participants ascribe specific values to their experiences, actions, and to their interplay with others. Things become meaningful to individuals when they understand their own way of sensing, thinking and acting. This can be achieved by connecting specific events and situations from their training to a broader understanding of how they see their physical activity. Telling certain stories about themselves and significant events they are involved in or plan to be helps in developing meaning.

Each subject, randomized to this intervention, is offered 5 coaching sessions (30 - 45 min.) primarily during working hours. The sessions will be conducted as group coaching with a maximum of 12 subjects attending each session.

The aims of the coaching sessions are 1) to motivate the workers to participate in the training sessions, 2) to make the workers establish and maintain healthy lifestyles, and 3) to assist the workers in encouraging their colleagues to join the physical training sessions and the coaching sessions.

The first coaching session will primarily focus on mutual expectations and motivation for doing physical exercise. The following sessions will primarily focus on issues brought up by the participants such as motivation, values around exercise, goals, healthy lifestyle, ideal training conditions, lack of support from colleagues, how to get colleagues to participate, and how to maintain an exercise routine.

The coaching is conducted according to principles from 3rd generation coaching and evidence-based coaching. The coaches 1) will drew on the best available knowledge from research and practice in coaching, exercise and sports, 2) integrate the knowledge into their individual coaching styles, and 3) adapt the coaching to fit the participants and the context. The coaches are students participating in courses at the Coaching Psychology Unit at University of Copenhagen, Denmark.

### Physical exercise intervention at home

Subjects randomized to this group (n = 89 subjects, n = 9 clusters) will perform physical exercises during leisure time at home. We have prioritized a training program design that is cost-efficient and involves easy-to-use exercises and training equipment based on the assumption that subsequent post-intervention implementation at home will only occur if the program is easily adopted and transparent.

The subjects will receive a bag with; 1) training equipment (easy-, medium- and hard- elastic tubing) and 2) 3 posters that visually demonstrate the exercises for the shoulder-, abdominal- and back muscles and contained recommendations for training progression. Poster one [37] illustrates 5 exercises for the back, shoulder and arm using elastic tubing and specially designed for clinical workers. The exercises are: 1) reverse flys, 2) shoulder squeeze, 3) shoulder external rotation, 4) wrist extension and 5) wood choppers. Poster two [38] illustrates 4 exercises for the back, shoulder and arm using elastic tubing. The exercises are: 1) shoulder raise, 2) shoulder squeeze, 3) shoulder rotation and 4) wrist extension. Poster three [39] illustrates 4 exercises for the back and abdominal muscles. The exercises are: 1) pelvic tilt, 2) quadruped leg/arm raise, 3) side plank and 4) lean and turn. As long as the participants perform the exercises during leisure time (e.g. at home) they are welcome to train with their colleagues, but they are not specifically encouraged to do so.

#### Ergonomic training and education

During the period of intervention the participants of each group is offered courses with ergonomic training and education in patient transfer and use of assistive devices. The courses are offered by the hospital’s working environment department.

### Blinding

Due to the interventional trial design, participants and instructors at the workplace cannot be blinded to group allocation. However, outcome assessors and data analysts will be blinded to group allocation.

### Outcome measures

Outcomes are measured by trained clinical examiners and by questionnaire survey at baseline and after the 10-week intervention period.

#### Primary outcome measures

The primary outcome is the change [at the individual level] from baseline to 10-week follow-up in average musculoskeletal pain intensity during the last week (average of back, neck and shoulder). Pain intensity was rated subjectively using a 0-10 modified VAS scale, where 0 indicates “no pain at all” and 10 indicate “worst pain imaginable” [26,40]. The body regions were defined by drawings from the Nordic questionnaire [41].

#### Secondary outcome measures

Secondary outcome measures are perceived exertion during work, work ability, social capital, back muscle reflex perturbation, postural control and maximal muscle strength and function of the back and shoulder muscles [at the individual level]. The strength tests are a part of the physical examination at baseline and follow-up.

### Sample size

A priori power analysis based on previous measurements revealed that 64 participants of each group for 95% power, SD of 1.5 and a minimal relevant difference of pain intensity of 1 [42] was sufficient to test the null-hypothesis of equality (α = 0.05). At an estimated 25% drop-out during the intervention period, group sizes were calculated to be at least 80. Due to an estimated inflation factor of 1.2 due to clustering effects, the estimated minimal group size should then be 96.

### Statistical analysis

All statistical analyses will be performed using the SAS statistical software for Windows (SAS Institute, Cary, NC). The change in pain (0-10 scale) will be evaluated using a repeated-measures two-way analysis of variance (ANOVA) with *group*, *time* and *group by time* as independent variables. Participant is entered as a random effect. Analyses will be adjusted for age and pain intensity at baseline. We will perform all statistical analyses in accordance with the intention-to-treat principle using a Mixed model approach which inherently accounts for missing values. An alpha level of 0.05 will be accepted as significant. Outcomes will be reported as between-group least mean square differences and 95% confidence intervals from baseline to follow-up.

## Ethics and dissemination

The study was approved by The Danish National Committee on Biomedical Research Ethics (The local ethical committee of Frederiksberg and Copenhagen; H-3-2010-062) as part of the research program “*Implementation of physical exercise at the workplace (IRMA)*”. The trial *“Implementation of Physical Exercise at the Workplace (IRMA08) - Healthcare Workers”* was registered in ClinicalTrials.gov (NCT01921764) prior to enrolment of participants.

The findings of this study will be presented at international conferences and published in peer-reviewed journals.

## Discussion

In this cluster randomized trial we will investigate the change in the self-rated average pain intensity in the back, neck and shoulder and the associated perceived exertion during work after either 10 weeks of physical exercise at the workplace or at home. Cluster randomization was chosen to increase adherence by keeping the participants within their departments and therefore to avoid contamination between the individuals of the two interventions.

The reason for providing coaching sessions and instructors to the workplace-based intervention and not to the home-based intervention was to examine a study design with two contrasting interventions; one intervention that is performed at home (during leisure time) versus one intervention that is performed during working hours. Performing physical exercise at the workplace together with colleagues may be more motivating for some employees and thus increase adherence. On the other hand, physical exercise performed during working hours at the workplace may be costly for the employers in terms of time spend. Thus, relevant ground exists to compare the efficacy of workplace- versus home-based exercise on musculoskeletal pain.

The present study will provide documentation to better guide workplace initiatives to reduce musculoskeletal pain among employees with high force loadings as during patient transfer.

## Competing interests

The authors of the article declare that they have no conflict of interest what so ever. Further, the research has not received any funding or grant from any commercial source.

## Authors’ contributions

MDJ and LLA conceived the idea and design of the project and all authors participated in the methodologically development. MDJ, ES, MBP, AZK and KJ performed the clinical examination. EL and RS supervised the coaches. All authors approved and critically reviewed the final version of the manuscript.

## Pre-publication history

The pre-publication history for this paper can be accessed here:

http://www.biomedcentral.com/1471-2474/15/119/prepub
